# Atrial fibrillation episode patterns as predictor of clinical outcome of catheter ablation

**DOI:** 10.1007/s11517-022-02713-x

**Published:** 2022-11-21

**Authors:** Javier Saiz-Vivó, Valentina D. A. Corino, Alba Martín-Yebra, Luca T. Mainardi, Robert Hatala, Leif Sörnmo

**Affiliations:** 1 Medtronic: Bakken Research Center, Maastricht, The Netherlands; 2 Department of Electronics, Information and Bioengineering, Politecnico Di Milano, Milan, Italy; 3 CIBER in Bioengineering, Biomaterials & Nanomedicine, Zaragoza, Spain; 4 BSICoS Group, I3A, IIS Aragón, Universidad de Zaragoza, Zaragoza, Spain; 5 Department of Arrhythmias and Cardiac Pacing, National Institute of Cardiovascular Diseases, Bratislava, Slovakia; 6 Department of Biomedical Engineering, Lund University, Lund, Sweden

**Keywords:** Atrial fibrillation, Ablation, Recurrence, Temporal aggregation, AF burden

## Abstract

Methods for characterization of atrial fibrillation (AF) episode patterns have been introduced without establishing clinical significance. This study investigates, for the first time, whether post-ablation recurrence of AF can be predicted by evaluating episode patterns. The dataset comprises of 54 patients (age 56 ± 11 years; 67% men), with an implantable cardiac monitor, before undergoing the first AF catheter ablation. Two parameters of the alternating bivariate Hawkes model were used to characterize the pattern: AF dominance during the monitoring period (log(mu)) and temporal aggregation of episodes (beta1). Moreover, AF burden and AF density, a parameter characterizing aggregation of AF burden, were studied. The four parameters were computed from an average of 29 AF episodes before ablation. The risk of AF recurrence after catheter ablation using the Hawkes parameters log(mu) and beta1, AF burden, and AF density was evaluated. While the combination of AF burden and AF density is related to a non-significant hazard ratio, the combination of log(mu) and beta1 is related to a hazard ratio of 1.95 (1.03–3.70; *p* < 0.05). The Hawkes parameters showed increased risk of AF recurrence within 1 year after the procedure for patients with high AF dominance and high episode aggregation and may be used for pre-ablation risk assessment.

**Graphical Abstract:**

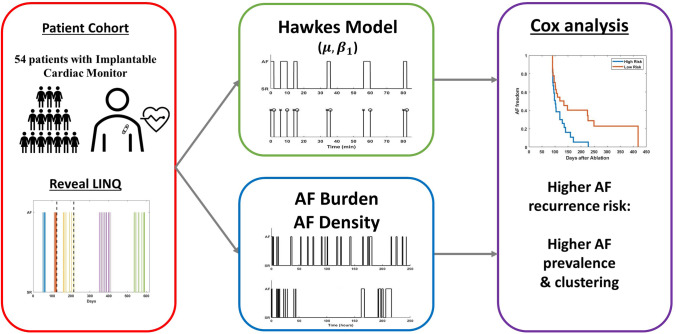

## Introduction

Atrial fibrillation (AF) is a progressive disease often initially manifested by intermittent episodes terminating spontaneously and eventually leading to sustained forms of AF for a subset of patients [1]. Over the decades, catheter ablation, and more specifically, pulmonary vein isolation (PVI), has become a common treatment of AF patients, especially for those whose antiarrhythmic drug therapy was inefficient (or not tolerated) for rhythm stabilization [2, 3] or those who were highly symptomatic [4]. However, long-term efficacy of catheter ablation reported in AF single-procedures does not exceed 70% [5]. Studies investigating risk factors associated with AF such as well-established scoring systems, including thromboembolic risk predictors like CHADS_2_ or CHA_2_DS_2_-VASc [6], and specific rhythm outcome predictors such as APPLE [7], SUCCESS [8], or MB-LATER [9], have focused on whether AF is present or absent. With the development of long-term monitoring devices, the binary approach is slowly being replaced by an approach involving features such as AF burden, i.e., the percentage of time in AF [10] which has been found to be a significant predictor of patients at risk of ischemic stroke [11]. Nonetheless, this measure does not describe whether AF episodes are clustered or distributed evenly throughout the monitoring period despite that characterization of the episode pattern may be relevant for better understanding of AF progression and risk assessment of AF recurrence post-ablation.

Implantable cardiac monitors (ICMs) offer the advantage of continuous long-term monitoring and can therefore be used to characterize episode patterns spanning long monitoring times. Such characterization has mainly focused on statistical analysis of either interepisode intervals, i.e., the interval between consecutive AF episodes [12–14] or inter-detection intervals, i.e., the interval between the onset of consecutive AF episodes [15], without accounting for episode history in the analysis. While these descriptive studies speculated that the information on episode patterns could be useful to predict AF recurrence [16], the clinical significance has not yet been established.

Recently, the alternating bivariate Hawkes model, a novel statistical approach to characterizing AF episode patterns, was proposed where episodes are assumed to be history-dependent [17]. In the present work, the performance of a subset of the model parameters is evaluated to predict the risk of AF recurrence. In addition, the performance of AF burden and AF density, being one of the very few parameters proposed for characterizing the temporal aggregation of the daily AF burden in patients using an ICM [18], is evaluated. To the best of our knowledge, there have been no studies using this or any other episode pattern characterization method as AF recurrence risk predictor.

## Methods

### Alternating bivariate Hawkes model

A statistical approach to characterizing episode patterns in paroxysmal AF (PAF) is based on history-dependent point process modelling of the transition times from sinus rhythm (SR) to AF and vice versa [17]. With the bivariate Hawkes model, the episode pattern is modelled by two alternating point processes{${N}_{1}\left(t\right),{N}_{2}\left(t\right), t>0$} which describe the number of transitions that have occurred up to $t$.: one accounting for transitions from SR to AF occurring at times (points) ${t}_{\mathrm{1,1}}, {t}_{\mathrm{1,2}},\dots$, and another for transitions from AF to SR occurring at times ${t}_{\mathrm{2,1}}, {t}_{\mathrm{2,2}},\dots$; the first subscript describes the type of transition (SR-to-AF AF-to-SR are denoted 1 and 2, respectively) and the second, the transition number. For simplicity in this study, SR and AF are assumed to be the alternating rhythms with only AF interrupting a SR rhythm and vice versa, while, in practice, a non-AF rhythm may very well replace SR.

The counting processes ${N}_{1}\left(t\right)$ and ${N}_{2}\left(t\right)$ are defined by two conditional intensity functions of the form [19]:

$${\mathrm\lambda}_{\mathrm m}\left(\mathrm t\right)={\mathrm\mu}_{\mathrm m}+\sum\limits_{\mathrm n=1}^2\sum\limits_{\left\{\mathrm k:\mathrm t>{\mathrm t}_{\mathrm n,\mathrm k}\right\}}{\mathrm\alpha}_{\mathrm m,\mathrm n}\mathrm e^{-{\mathrm\beta}_{\mathrm m,\mathrm n}\left(\mathrm t-{\mathrm t}_{\mathrm n,\mathrm k}\right)}$$

where ${\mu }_{m}>0$, ${\alpha }_{m,n}$ ≥ 0 and ${\beta }_{m,n}\ge 0$ for $m, n=1, 2$. The main characteristic of the model is that the conditional intensity function ${\lambda }_{1}(t)$ increases by ${\alpha }_{\mathrm{1,1}}$ immediately after an SR-to-AF transition (self-excitation) and then decreases exponentially, defined by the decay parameter ${\beta }_{\mathrm{1,1}}$, to the base intensity ${\mu }_{1}$ reflecting the mean rate of SR-to-AF transitions. The conditional intensity function ${\lambda }_{2}(t)$ characterizes AF-to-SR transitions and behaves similarly to ${\lambda }_{1}(t)$, defined by the excitation parameter ${\alpha }_{\mathrm{2,2}}$, the decay parameter ${\beta }_{\mathrm{2,2}}$, and the base intensity ${\mu }_{2}$. As the probability of additional transitions increases immediately after a transition, the process can account for aggregation behaviour. In addition to the self-excitation, both ${\lambda }_{1}(t)$ and ${\lambda }_{2}(t)$ contain additional terms, defined by ${\alpha }_{\mathrm{1,2}}$ and ${\beta }_{\mathrm{1,2}}$ in the case of ${\lambda }_{1}(t)$, and by ${\alpha }_{\mathrm{2,1}}$ and ${\beta }_{\mathrm{2,1}}$ in the case of ${\lambda }_{2}(t)$, which lets the counting processes influence each other (cross-excitation).

The bivariate Hawkes model in its original formulation does not impose alternating transitions; i.e., an SR-to-AF transition is not necessarily followed by an AF-to-SR transition, while from the physiological point of view, this is required. This disadvantage is eliminated by multiplying ${\lambda }_{1}(t)$ and ${\lambda }_{2}(t)$ with a binary “occurrence” function

$${\mathrm o}_1\left(\mathrm t\right)=\left\{\begin{array}{c}1,\;\;\;\;\;\;\;{\mathrm N}_1\left(\mathrm t\right)={\mathrm N}_2\left(\mathrm t-{\mathrm d}_2\right),\\0,\mathrm{otherwise},\end{array}\right.$$

and

$${\mathrm o}_2\left(\mathrm t\right)=\left\{\begin{array}{c}1,\;\;\;\;\;\;{\mathrm N}_2\left(\mathrm t\right)\neq{\mathrm N}_1\left(\mathrm t-{\mathrm d}_1\right),\\0,\mathrm{otherwise}.\end{array}\right.$$

which ensures that AF occurs after SR, and that SR occurs after AF, respectively.

The parameters ${d}_{1}$ and ${d}_{2}$ define the minimum duration of AF and SR, respectively.

Finally, the conditional intensity functions for the alternating, bivariate Hawkes process are given by:

$${\widetilde{\mathrm\lambda}}_{\mathrm m}\left(\mathrm t\right)={\mathrm\lambda}_{\mathrm m}\left(\mathrm t\right){\mathrm o}_{\mathrm m}\left(\mathrm t\right),\mathrm m=1,2.$$

The structure of ${\widetilde{\lambda }}_{m}\left(t\right)$ is identical to that of the bivariate Hawkes process ${\lambda }_{m}\left(t\right)$, except that a SR-to-AF transition can, once a certain time ${d}_{1}$ has elapsed, only be followed by an AF-to-SR transition, and so on.

**Fig. 1:**
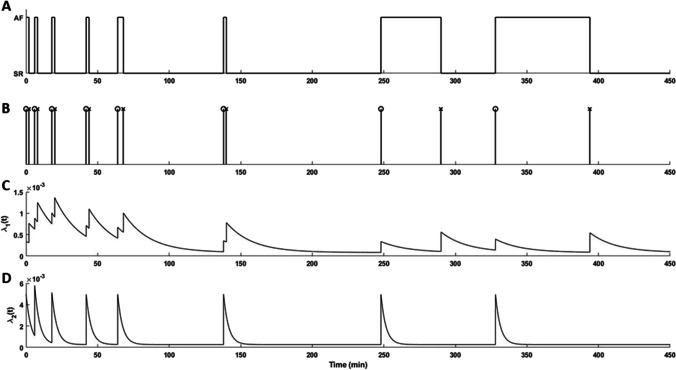
**A** Example of real AF episode pattern. **B** Transition times for the episode pattern in **A**. The marks “o” and “x” indicate SR-to-AF and AF-to-SR transitions, respectively. **C**–**D** The conditional intensity function of SR-to-AF transitions and of AF-to-SR transitions. For reasons of clarity, ${{\lambda}}_{1}\left({t}\right)$ and ${{\lambda}}_{2}\left({t}\right)$ are displayed rather than ${\widetilde{{{\lambda}}}}_{1}\left({{t}}\right)$ and ${\widetilde{{{\lambda}}}}_{2}\left({{t}}\right)$

Figure 1 shows an AF episode pattern, the transition times corresponding to the two alternating point processes and the conditional intensity functions associated to those point processes.

The model parameters, defining the conditional intensity functions, can be estimated using the maximum likelihood (ML) method. For a bivariate process, the likelihood function is given by [20]:

$${ln} \mathcal{L}\left({\boldsymbol{{\theta}}};{\bf{t}}\right)= \sum\limits_{m=1}^{2}\sum\limits_{k=1}^{{N}_{m}(T)}{ln}{\lambda }_{m}({t}_{m,k};{\boldsymbol{\theta}})-\boldsymbol{ }\sum\limits_{m=1}^{2}{\int }_{0}^{T}{\lambda }_{m}\left({\bf{t}};{\boldsymbol{\theta}}\right)dt$$

where the vector $\bf {{t}}$ contains the transition times in the observation interval [0, T] and the vector ${\boldsymbol{\theta}}$ collects all the model parameters, i.e., ${\boldsymbol {\theta}}=[{\mu }_{1}, {\mu }_{2},{\alpha }_{\mathrm{1,1}},{\beta }_{\mathrm{1,1}},{\alpha }_{\mathrm{1,2}},{\beta }_{\mathrm{1,2}},{\alpha }_{\mathrm{2,1}},{\beta }_{\mathrm{2,1}},{\alpha }_{\mathrm{2,2}},{\beta }_{\mathrm{2,2}}]$. The ML estimator is given by:

$$\boldsymbol{ \widehat{{{\theta}}}}=\mathrm{arg}\underset{\boldsymbol\theta }{\mathrm{max}}(\mathrm{ln}\mathcal{L}\left({{\boldsymbol\theta}};{\boldsymbol{t}}\right))$$

see [17] for details of the ML estimator.

It is then assumed that ${\beta }_{\mathrm{1,1}}= {\beta }_{\mathrm{1,2}}= {\beta }_{1}$ and ${\beta }_{\mathrm{2,1}}= {\beta }_{\mathrm{2,2}}= {\beta }_{2}$. Hence, the conditional intensity functions are defined by a relatively small number of parameters and therefore suitable for statistical inference. The onset of the first AF episode and the end of the last AF episode are assumed to be entirely contained in the observation interval. Thus, the first transition for analysis is from SR-to-AF and the last from AF-to-SR.

**Fig. 2:**
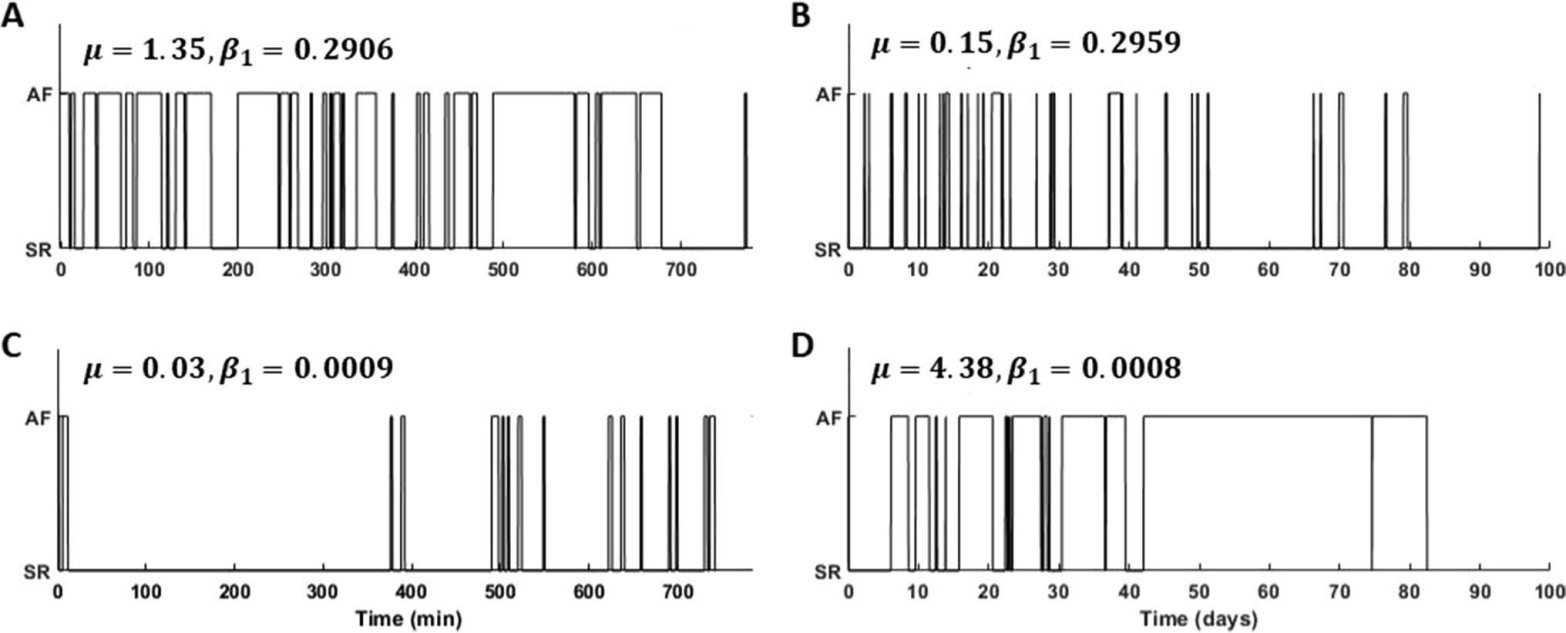
Episode patterns and the estimated Hawkes model parameters μ and β_1_. **A** and **C** are short segments around 800 min with **A** episode pattern dominated by AF with a lower degree of aggregation (β_1_≈0.3) and **C** episode pattern dominated by SR with a higher degree of aggregation (β_1_≈0). **B** and **D** are long segments up to 100 days with **B** episode pattern dominated by SR and a lower degree of aggregation and **D** episode pattern dominated by AF and a higher degree of aggregation

The base intensity ratio is defined as:

$$\mu =\frac{{\mu }_{1}}{{\mu }_{2}}$$

and provides information on the dominating rhythm of the analysed segment: $\mu >1$ indicates dominance of AF (Fig. 2A and D) and $\mu <1$ dominance of SR (Fig. 2B and C). In the present study, the natural logarithm of $\mu$ ($\mathrm{log}(\mu )$) is used instead of $\mu$ as $\mu$ is a ratio, and, therefore, $\mathrm{log}(\mu )$ exhibits a more linear behaviour.

The decay parameter ${\beta }_{1}$, empirically restricted to a range between 0 and 0.3 [17], describes the degree of episode aggregation, where a value of ${\beta }_{1}$ close to 0.3 reflects few clusters. This is illustrated in Fig. 2A and B where the episodes are spread out throughout the monitoring period, although the time span differs considerably (A in minutes and B in days). Conversely, a value of ${\beta }_{1}$ close to 0 reflects high episode aggregation as illustrated in Fig. 2Cand D. In this study, ${\beta }_{2}$ is not considered for prediction as ${\beta }_{1}$ is deemed to play the main role with regard to AF episode aggregation.

The Hawkes model requires a minimum number of episodes to produce adequate parameter estimates, here set to 10, i.e., 20 transitions, as suggested in [17].

For further details on the alternating bivariate Hawkes model and the estimation of ${\mu }_{1}$, ${\mu }_{2}$ and ${\beta }_{1}$, the reader is referred to [17].

### AF density

AF density is defined as the ratio of the cumulative deviation of the patient’s actual AF burden level from a hypothetical uniform burden level, to that of the hypothetical maximum burden aggregation [18].

For a patient with a total AF burden $b$ (expressed as the proportion of the observed time $T$ in which a patient is in AF), the patient’s AF burden level corresponds to:

$$\mathrm F\left(\mathrm p;\mathrm b\right)=\frac{\mathrm T(\mathrm p;\mathrm b)}{\mathrm T}$$

with $T(p;b)$ denoted as the minimum continuous period required for the development of a proportion $p$ of the patient’s total observed burden $(b)$.

The cumulative deviation of the patient’s actual burden from a hypothetical uniform burden level can be evaluated as:

$${\int }_{0}^{1}\left|{F}^{^{\prime}}\left(p;b\right)-p\right|dp$$

where *F’*(*p;b*) is the patient’s actual burden, and *F*(*p;b*) = *p* corresponds to the hypothetical uniform burden level defined by the burden evenly distributed throughout the monitoring period.

Conversely, the hypothetical maximum burden aggregation is defined by the total burden comprised in one continuous episode and simplified to:

$$\frac{\left(1-b\right)}{2}$$

**Fig. 3:**
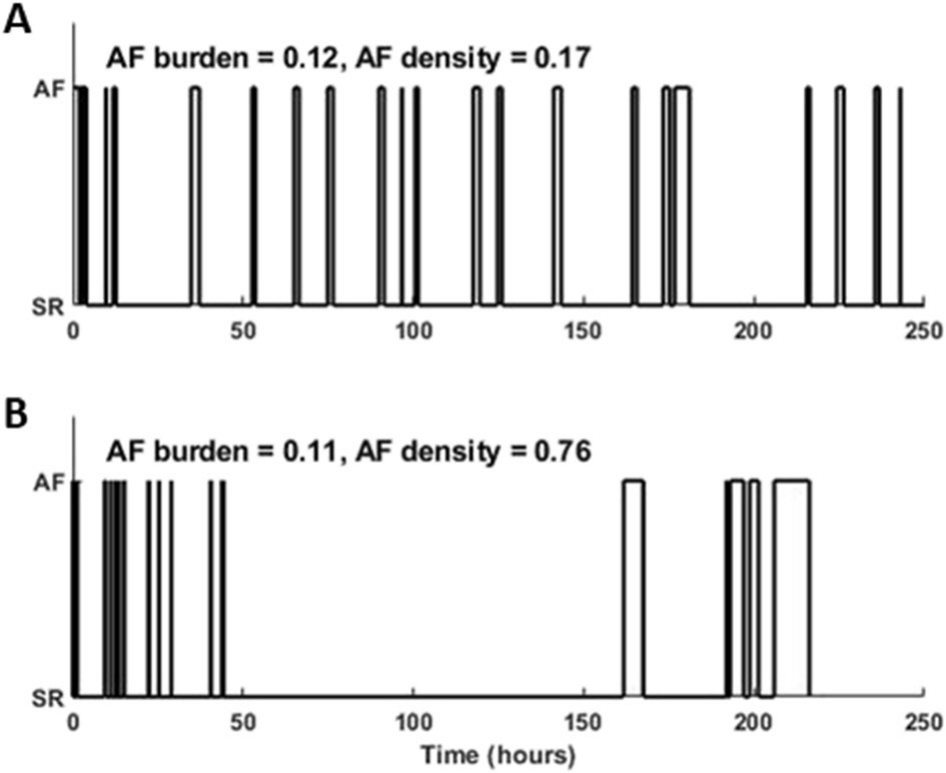
Patients with different types of temporal aggregation but with similar AF burden (≈ 0.12) with **A** low aggregation (AF density = 0.17) and **B** high aggregation (AF density = 0.76)

Finally, the AF density is defined as:

$$\mathrm{AF}\;\mathrm{density}=2\frac{\int_0^1\left|\mathrm F\left(\mathrm p;\mathrm b\right)-\mathrm p\right|\mathrm{dp}}{1-\mathrm b}$$

and assumes values on the interval [0,1], where a value close to 0 indicates a homogeneous distribution of AF burden, whereas a value close to 1 indicates that AF burden is confined to an interval much shorter than the monitored period. Figure 3 shows examples of temporal aggregation for two patients with similar levels of AF burden and monitoring time, with low and high temporal aggregation.

### Cohort

The Reveal LINQ USABILITY study (ClinicalTrials.gov Identifier: NCT01965899), a multi-centre single-arm clinical study [21], was merged with a database from the National Institute of Cardiovascular Diseases in Bratislava, Slovakia [22]. The patients of both cohorts, with documented history of AF and ablation candidates, provided written informed consent, and the study protocols were reviewed and approved by the Human Research Ethics Committee of each participating institution.

Out of the 226 enrolled patients, 99 had pre-ablation data, out of which 19 were excluded due to previously failed ablation and 26 had less than 10 episodes before catheter ablation (the minimum number of episodes required by the model). Therefore, the analysis included 54 patients (age 56 ± 11 years; 67% men) with a documented history of AF (74% PAF, the remaining being persistent AF), and ablation candidates.

The baseline and clinical characteristics of the study cohort are shown in Table 1.

**Table 1:** Baseline and clinical data of the study population (*n* = 54). PAF, paroxysmal atrial fibrillation; CAD, coronary artery disease

Patient characteristic	
Age (years)	56 ± 11
Male gender (%)	36 (67%)
Coronary risk profile	
PAF	40 (74%)
Hypertension	21 (39%)
Diabetes	7 (13%)
CAD	3 (5%)
Stroke	3 (6%)

### Data collection

The ICM was implanted 2.7 (1–15) months, median (min–max), before the ablation procedure, and the patients had 10.9 (3–4) months follow-up for AF recurrence detection. AF recurrence was defined as an AF episode detected by the ICM after a 3-month blanking period following catheter ablation. The blanking period is based on reports on the efficacy of catheter ablation describing how early recurrences could be caused by post-ablation inflammation or short-term autonomic imbalance rather than ablation failure [23].

The devices used in the usability and the Slovakia studies were the Reveal LINQ and Reveal XT (Medtronic Inc, Minneapolis, MN), respectively, implanted within the fourth intercostal space (V2–V3 electrode orientation). Both devices sense and detect the rhythm and store the onset and duration of the AF episodes. The AF detection algorithm is based on an R-R interval pattern-based algorithm and a *P*-wave evidence score which reduces false positive AF detections and leverages the evidence of a single *P*-wave between two *R* waves using morphologic processing of the ECG signal. The algorithm makes a rhythm classification every 2 min [24]. This provides us the values for ${d}_{1}$ and ${d}_{2}$, the minimum duration of AF and SR, respectively. The data stored in the device is downloaded each time the patient has a clinical appointment. However, due to memory restrictions, the device can store up to 30 episodes of data, with the new episode overwriting the oldest one. Therefore, only data from the last 30 episodes before each data download were available. Nonetheless, several downloads could be grouped together if temporal continuity existed between them, increasing the number of episodes available for characterization.

**Fig. 4:**
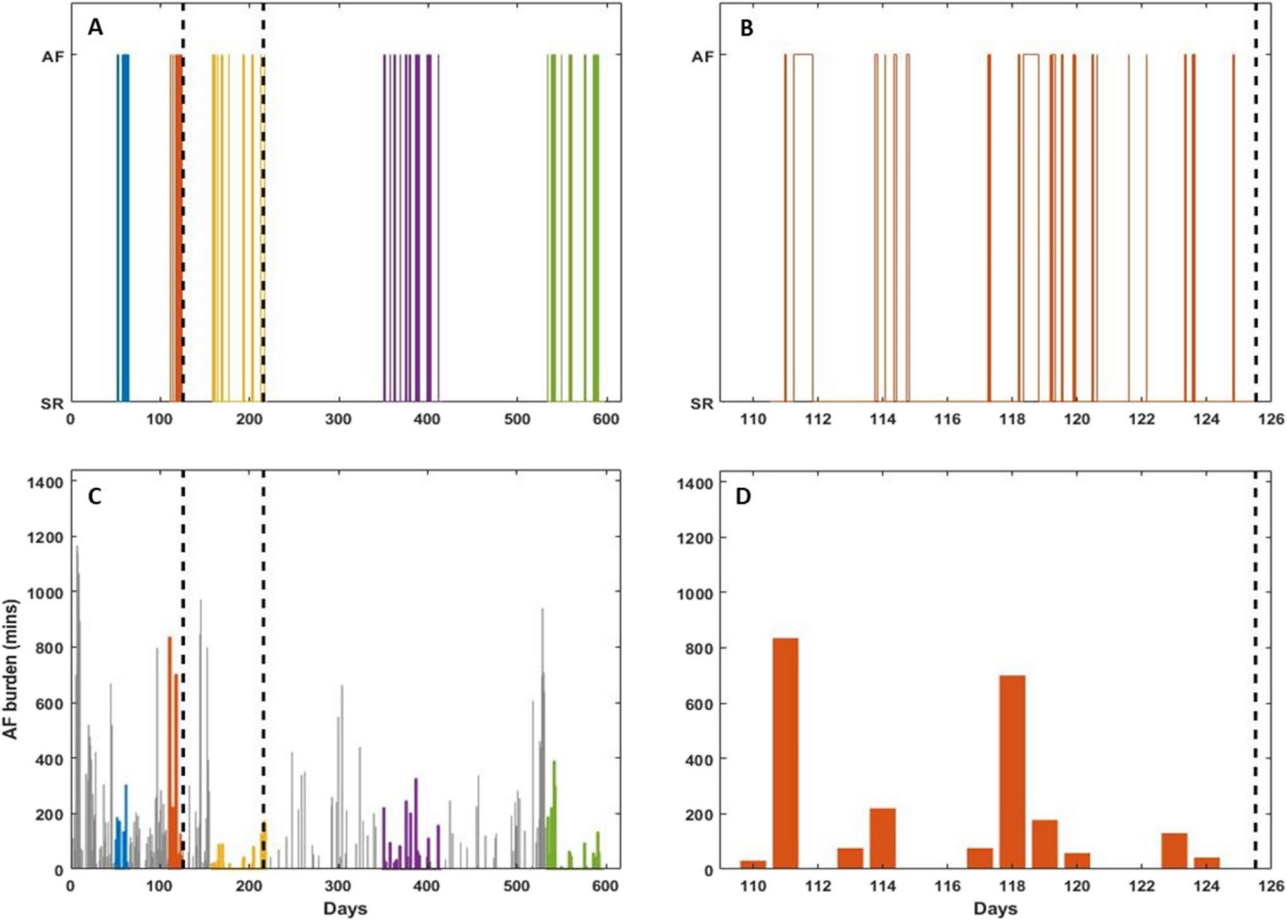
Example of data extracted from Reveal LINQ/XT presented as a function of days of monitoring; the leftmost dashed line marks the catheter ablation, and the following dashed line marks the end of the 3-month blanking period. **A** Episodes with onset and duration downloaded in five sessions (blue, orange, yellow, purple, and green). **B** Episodes with onset and duration downloaded from the last session before catheter ablation (second session in **A**). **C** Daily AF burden detected in minutes (grey) with highlights on the days where the episodes have onset and duration information (color-coded as in **A**). **D** Daily AF burden detected in minutes during the last session before catheter ablation

In addition to the onset and duration of each AF episode, the device stored the daily AF burden in minutes for the entire monitoring period. An example of data extracted from the device is presented in Fig. 4 with the ablation date and the end of the 3-month blanking period marked by dashed lines. It also shows the rhythm condition (either SR or AF) of the patient extracted from each session (color-coded) where the onset and duration of the episodes can be derived, and the daily AF burden stored for the entire monitoring period highlighting AF burden during the stored sessions, as well as the rhythm condition of the last session before catheter ablation and its corresponding daily AF burden.

### Statistical analysis

The four parameters log(μ), β_1_, AF burden, and AF density were computed using the episode information of the last available session before catheter ablation. Continuous data are presented as mean ± standard deviation if the null hypothesis H_0_ of the Kolmogorov–Smirnov test (H_0_: data is normally distributed) was not rejected. Otherwise, continuous data are presented as median (min–max). Categorical data are presented as absolute frequency (relative frequency in percentage).

The primary endpoint (time to AF recurrence) was analysed using the Kaplan–Meier method, and the null hypothesis was tested by means of the log-rank test. The hazard ratio (HR) and its confidence intervals were computed using Cox’s proportional hazards models. For the one-parameter prediction, the patients were dichotomized into high- and low-risk groups based on the optimal cut-off value chosen to maximize the separation between groups. This was accomplished by evaluating the Cox proportional hazard regression in the different groups of patients divided by a threshold that varied from quantile 25–75% with 5% increments. The regression with the lowest *p*-value was selected as the optimum separation cut-off. In case of β_1_, the parameter was found to be bimodal so the cut-off was selected as the average between the lower limit (β_1_ = 0) and the upper limit (β_1_ = 0.3) In the two-parameter prediction, a linear combination of the selected parameters and the corresponding regression coefficients in the Cox model was computed and high- and low-risk groups were defined, based on the median of the combination. The Hawkes combination, defined by log(µ) and β_1_, provides information on dominating rhythm (AF or SR) and episode aggregation. Similarly, the combination of AF burden and AF density provides information on dominating rhythm and episode aggregation. The null hypothesis was rejected when *p* < 0.05, then set as the level of significance. The statistical analysis was performed using Matlab R2019b (The Mathworks Inc., Natick, Massachusetts).

## Results

During the monitoring period before ablation, the patients had between 1 and 4 data downloads with 96 (20–188) days between scheduled appointments. For the present analysis, the focus was set on the last data download before ablation which in 43 (80%) patients occurred 1 month before ablation (75% during the last week) and contained 29 (10–37) AF episodes within a monitoring period of 9.8 (23.3) days, ranging from 2.6 h to 7 months.

The relationship between the parameters was explored, and out of the considered variables, only AF burden showed high correlation with log(μ) (*r* = 0.78; *p* < 0.001). Even though both β_1_ and AF density reflect different aspects of episode aggregation, they were found to be weakly correlated (*r* =  − 0.07; *p* = 0.63), and, therefore, may provide complementary information. When studying the distribution of β_1_, it was found to be bimodal showing that AF episodes were either highly clustered or uniformly distributed throughout the monitoring period.

In the analysed cohort, 41 patients (76%) had AF recurrence within 15 months following catheter ablation and the overall estimated event-free rate at 1 month after the blanking period (4 months after catheter ablation) was 39%. The statistical analysis of the parameters extracted from the last data download shows that there are no statistical differences between patients having had AF recurrence and those not having (*p* > 0.05 for all parameters).

**Fig. 5:**
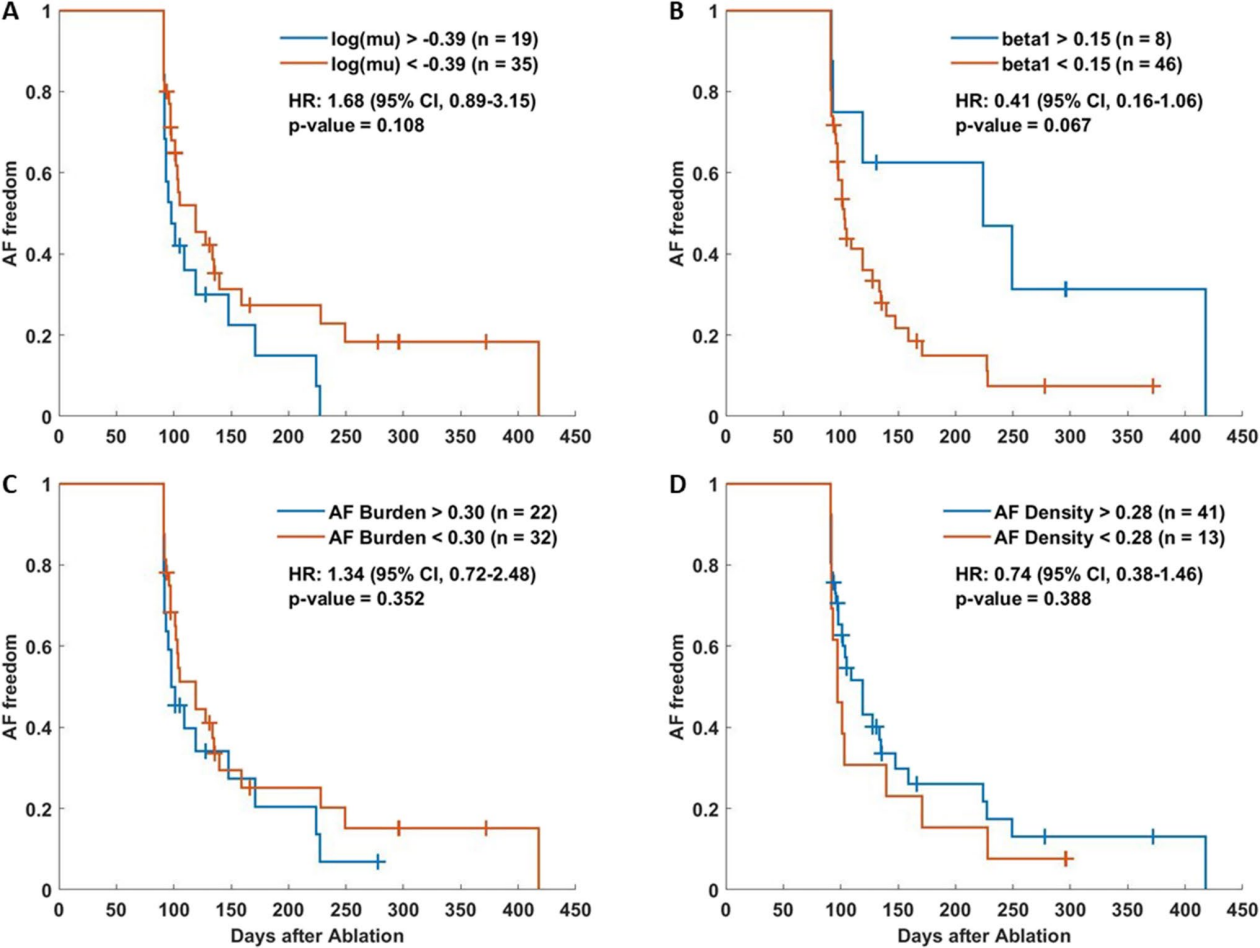
Kaplan–Meier curves for AF freedom after catheter ablation using each parameter as a risk predictor: **A** $\text{log}(\mu)$, **B** $\beta_{1}$ **C** AF burden, and **D** AF density. The legend of each panel shows the threshold used and the number of patients in each group, and the panels show the hazard ratio (HR) and the 95% confidence intervals with their significance levels. Plus signs symbolize the censored patients

The one-parameter analysis showed no significant differences (log-rank *p* > 0.05) between high- and low-risk groups for the selected parameters (Fig. 5).

In two-parameter Cox analysis, AF burden and AF density were linearly combined and weighted with their respective Cox coefficient (0.03 for AF burden and $-0.02$ for AF density). The positive coefficient indicates a positive effect of the covariate AF burden to the risk of AF recurrence, meaning that more AF would increase the risk of AF recurrence. Conversely, a negative coefficient for AF density indicates that a higher AF density, i.e., a higher episode aggregation, reduces the risk of AF recurrence.

**Fig. 6:**
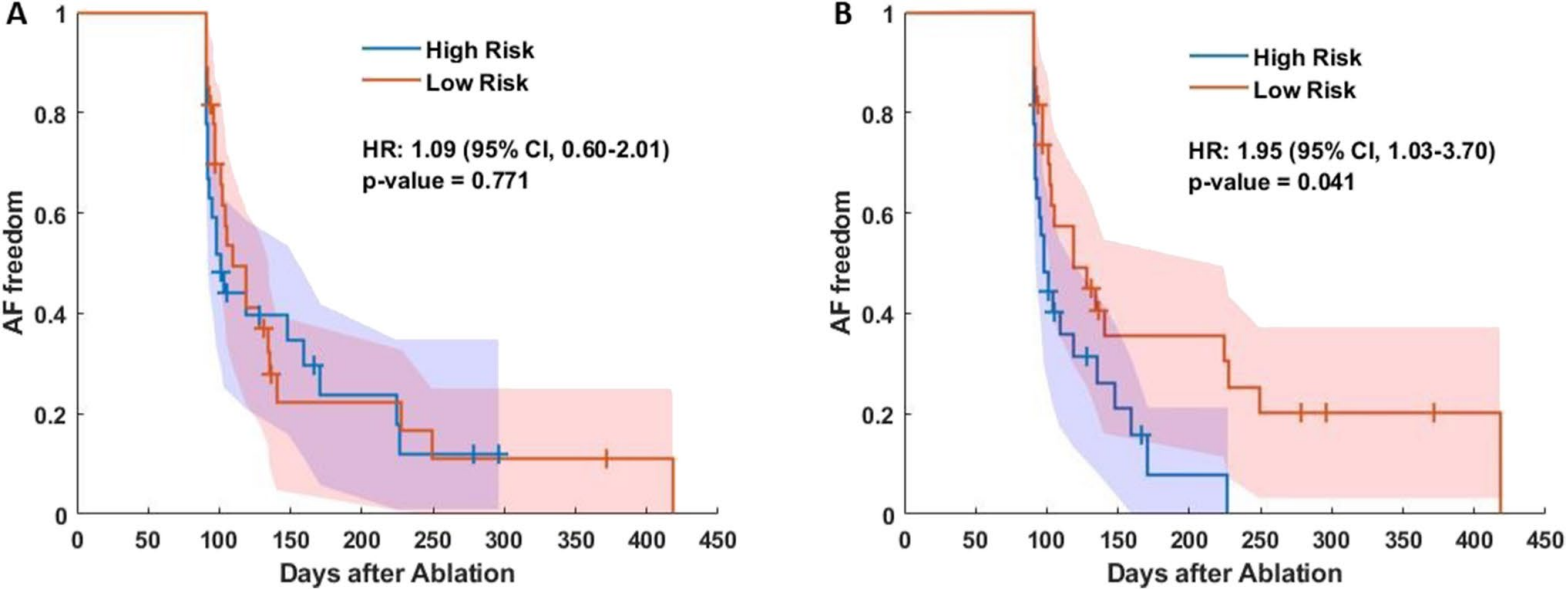
Kaplan–Meier and 95% confidence intervals curves for AF freedom after catheter ablation combining **A** AF burden and AF density, and **B** the Hawkes parameters. The panels show the hazard ratio (HR) and the 95% confidence intervals with their significance levels. Plus signs symbolize the censored patients

The combination of AF burden and AF density (Fig. 6A) is related to a 1.09 (95% CI, 0.60–2.01; *p* = 0.77) higher risk of early recurrence between the high- and low-risk groups (defined by the median value of the combination); however, the results are non-significant for this combination.

The parameters log(μ) and β_1_ were also linearly combined and weighted with their respective Cox coefficient (0.23 for log(μ) and –0.36 for β_1_). The positive effect of the covariate log(μ) to the AF recurrence risk indicates that a higher AF dominance would increase the risk of AF recurrence, while a negative coefficient for β_1_ indicates that a higher β_1,_, i.e., less episode aggregation, reduces the risk of AF recurrence.

In this case, the combination of log(μ) and β_1_ is associated with a higher risk of early AF recurrence with an HR of 1.95 (95% CI, 1.03–3.70; *p* < 0.05) (Fig. 6B). The estimated event-free rates at 1 month after the blanking period were 31% for high-risk patients and 49% for low-risk patients. In addition, 21 (78%) patients at high risk had AF recurrence, while 20 (74%) patients at low risk had AF recurrence (chi-squared *p* = 0.31). Even though both groups had similar proportions of AF recurrence, the survival times for the patients at high risk which had AF recurrence was less than 10 months while for those in the low-risk group was 14 months.

## Discussion

ICMs with high AF detection accuracy offer the unique advantage of long-term monitoring periods spanning several months which can lead to a more detailed characterization of AF behaviour. With the rapidly increasing use of these continuous monitoring devices for patients diagnosed with AF [25] and the relatively high recurrence rates post-catheter ablation [23], the need for a method to characterize AF episode patterns to evaluate the risk of recurrence is increasingly important. To the best of our knowledge, there have been no studies using episode pattern characterization method as AF recurrence risk predictor. Our approach is also the first one comparing different parameters to determine the risk of AF recurrence in a cohort of continuously monitored AF patients outside of the restrictions 24-h Holter devices entail.

In recent years, the problem of how to characterize episode patterns has received certain attention. However, it has been mainly focused on statistical analysis of either interepisode intervals, i.e., the interval between consecutive AF episodes [12–14] or inter-detection intervals, i.e., the intervals between onset of consecutive AF episodes [15]. The main drawback of this type of analysis is that it resides on the assumption that episodes are statistically independent, which may be questioned since AF episodes tend to cluster [13]. The alternating, bivariate Hawkes model was developed to provide a model-based, statistical approach to characterizing the dynamics of episode patterns [17]. While that work conjectured that the episode pattern could offer insight into AF and the degree of atrial electrical and structural remodelling, the clinical significance of log(μ) and β_1_ has not been established previously.

Numerous risk factors have been linked to recurrent AF after ablation, including thromboembolic risk predictors like CHADS_2_ or CHA_2_DS_2_-VASc [6] and other specific rhythm outcome predictors such as APPLE [7], SUCCESS [8], and MB-LATER [9]. These scores have shown limited risk evaluation capability and have the drawback of relying on the detection of AF recurrence in patients using conventional Holter devices and the need of image-based parameters such as ejection fraction or left atrial diameter. In particular, MB-LATER uses early recurrence of AF as a feature and therefore cannot be used to evaluate the risk of AF recurrence before attempting the catheter ablation procedure. Conversely, the proposed method uses a subset of parameters estimated from a model-based approach which characterizes AF episode patterns in a continuously monitored cohort of patients.

In the analysis of the recurrence predictors, no statistical differences were found between the recurrence and no recurrence groups. However, when studying β_1_, we found that a higher proportion of patients with AF recurrence had more clustered episodes, i.e., β_1_ close to 0 (90% vs 69%, chi-squared *p* = 0.724). Although this gives us a first indication that patients with more episode aggregation may have a higher risk of AF recurrence, the overall proportion of patients with more aggregation is also high (85%) and the population is biased towards patients with AF recurrence.

Unsurprisingly, when evaluating the relationships between covariates, log(μ) and AF burden had significant correlation (*r* = 0.78; *p* < 0.001) as both parameters provide information on AF dominance (log(μ) > 0 and AF burden > 0.5). However, β_1_ was weakly correlated with AF density, and, while both features describe the degree of episode aggregation, β_1_ extracted from a statistical model, and AF density being an ad hoc parameter, these parameters may provide complementary information.

The parameters studied were estimated from the episodes stored from the last session available before catheter ablation containing episodes with durations from 2.6 h to 7 months. To produce a more homogeneous results and taking advantage of the long monitoring periods of the Reveal, the multivariate analysis was also computed for the last 4 weeks before the ablation. A monitoring period of 4 weeks was chosen as it was the minimum pre-ablation period common for the cohort. In this case, only AF burden and AF density were computed due to that while the Reveal stores the daily AF suffered by the patient, the onset and duration of the individual episodes were unavailable. The combination of AF burden and AF density showed a non-significant HR of 1.00 (95% CI 0.55–1.84; *p* = 0.99). This result, combined with the non-significant result of the Cox analysis for AF burden and AF density estimated over the last session, suggests that both AF burden and AF density do not convey significant information for assessing the risk of AF recurrence in this cohort. While AF density has not been used to assess risk of AF recurrence before, AF burden levels were shown to be able to predict the risk of AF recurrence [26]. The study found a lower risk of AF recurrence with a lower pre-ablation AF burden in AF patients. However, a significant difference in risk was found between those patients lower than 1% AF burden and those with higher levels of AF burden. Our patient population has relatively higher AF burden levels as our cut-off threshold between groups was defined as 30%.

The risk of AF recurrence for the Hawkes parameters was found to have an HR of 1.95 (95% CI, 1.03–3.70; *p* < 0.05). The combination showed that the risk was significantly higher for patients with a higher AF prevalence during their monitoring period and with higher degree of episode aggregation.

The log(μ) and β_1_ parameters of episode aggregation may represent an early sign of transition from paroxysmal to persistent AF. The observed increased risk of arrhythmia recurrence once the novel criteria are present would be well in line with lower catheter ablation efficacy in patients with persistent forms of AF. If confirmed, this could be used as an early triaging mechanism pointing towards the need of accelerated referral for ablation.

To assess the possible link between our novel variables and established AF recurrence risk factors, the clinical parameters age, hypertension, and AF type were considered. The link between classical AF risk factors and success of ablation was systematically evaluated previously by Balk et al. [27]. The multivariate analysis showed that neither age, AF type, nor hypertension showed a significant association to ablation success. This association was further explored in our study where we analysed the risk of the clinical risk factors and found non-significant hazard ratios. Some of the reasons behind this result, however, could be the relatively young population included in the study (56 ± 11 years) or the under-representation of non-paroxysmal AF patients (26%).

The retrospective analysis carries certain limitations as, for example, it was based on a limited patient population from 2 different cohorts implanted with the Reveal LINQ ICM, which automatically detects AF episodes longer than 2 min. Therefore, episodes longer than 30 s, defined as AF episodes by the guidelines [3], but shorter than 2 min were undetected by the ICM. Furthermore, due to memory restrictions, only the onset and duration of the last 30 episodes detected by the ICM before each data download are stored. The 96 (41) days between scheduled appointments (and therefore between data downloads) potentially resulted in a loss of AF episodes that could have been used to better characterize the episode patterns. In addition, due to the retrospective nature of the study, the medication administered to each patient during the monitoring period was not available. Despite these drawbacks, the advantage of having continuous monitoring of the patients before and after ablation greatly outweighs the disadvantages of possible information loss due the device resolution or memory restrictions. Using the Hawkes model, at least 10 episodes, i.e., 20 transitions, should be available to produce adequate results [17]; hence, with 30 stored episodes, the requirement is fulfilled.

## Conclusion

The clinical relevance of AF episode pattern characterization using the alternating, bivariate Hawkes model is evidenced by its capability to predict AF recurrence post-catheter ablation. The proposed parameter combination is related to increased risk of AF recurrence within 1 year of the procedure for patients with more dominant AF and more episode aggregation. This approach represents a preliminary step to demonstrate the clinical significance of AF episode pattern characterization as well as to popularize pre-ablation risk assessment which could be used in a more effective patient triage and reduce the economic and personal burden associated with the procedure.
